# Study and Experimental Validation of the Functional Components and Mechanisms of *Hemerocallis citrina* Baroni in the Treatment of Lactation Deficiency

**DOI:** 10.3390/foods10081863

**Published:** 2021-08-12

**Authors:** Jing Zhong, Yuxuan Liang, Yongchun Chen, Jiawei Zhang, Xiaoying Zou, Jie Deng, Da Wang, Yuanming Sun, Meiying Li

**Affiliations:** College of Food Science, South China Agricultural University, Guangzhou 510642, China; 20182145038@stu.scau.edu.cn (J.Z.); 20181145005@stu.scau.edu.cn (Y.L.); 20172123002@stu.scau.edu.cn (Y.C.); 526815158@stu.scau.edu.cn (J.Z.); zouxiaoying97@stu.scau.edu.cn (X.Z.); dengjie@stu.scau.edu.cn (J.D.); 20172013009@stu.scau.edu.cn (D.W.); ymsun@scau.edu.cn (Y.S.)

**Keywords:** *Hemerocallis citrina* Baroni, lactation insufficiency, network pharmacology

## Abstract

The function of *Hemerocallis citrina* Baroni (daylily) on promoting lactation is reported in several ancient Chinese medicine books. However, nowadays, there is no conclusive data to support this statement. In this study, we investigated the effect of *Hemerocallis citrina* Baroni extract (HCE) on lactation insufficiency in chronic unpredictable mild stress (CUMS) dams and further explored the mechanism and functional components through network pharmacology. The results showed that HCE could increase the offspring’s weight, serum prolactin (PRL), and oxytocin (OT) level of CUMS dams. Network pharmacology analysis revealed that the facilitation of HCE on lactation is the result of the comprehensive action of 62 components on 209 targets and 260 pathways, among this network, quercetin, kaempferol, thymidine, etc., were the vital material basis, signal transducer and activator of transcription 3 (STAT3), mitogen activity protein kinase 1 (MAPK1), tumor protein P53 (TP53), etc., were the core targets, and the prolactin signaling pathway was the core pathway. In addition, verification test results showed that HCE regulated the abnormal expression of the prolactin signaling pathway, including STAT3, cyclin D1 (CCND1), MAPK1, MAPK8, nuclear factor NF-kappa-B p105 subunit (NFKB1), and tyrosine-protein kinase (JAK2). In conclusion, HCE exhibited a facilitation of lactation insufficiency, in which quercetin, kaempferol, thymidine, etc., were the most important material basis. The mechanism of this promotional effect is mediated by the prolactin signaling pathway in mammary gland.

## 1. Introduction

Breastfeeding is usually known as the ideal way of feeding infants [1]. However, only approximately 40% infants aged between 0–6 months were exclusively breastfed worldwide during 2015–2020 [2]. Several studies have focused on the contributor of this issue and they revealed that many mothers with lactation defects have suffered maternal depression [3,4]. In addition, an increasing number of research have demonstrated that lactation is closely connected with depression, inducing a less maternal behavior in dams, which is related to a decrease of oxytocin (OT) and prolactin (PRL) levels [5,6,7]. However, the mechanism is very complex, which cannot be fully explained by existing studies.

In the past few years, the use of oral drugs to treat lactation insufficiency was the most common treatment of choice; however, it was shown that they provoked some adverse effects on mothers, such as headache, dry mouth, and gastrointestinal disturbances [8]. On the other hand, dietary therapies have been demonstrated to be more favorable, since they cause less side effects, are easily available, and their efficiency has become the mainstay against lactation insufficiency, attracting scientists who devote great effort to researching. The ingredients of dietary therapies consist of multiple plants with special effects, such as a large active substance system. According to the traditional research method, it is important to analyze each single compound and to evaluate its activity individually, due to the difficulties and interferences that being part of the complex network generate. However, numerous studies showed that, in equality dose, the use of plant crude extracts was more effective than isolating specific compounds [9,10], indicating that the efficacy on treatments is the result of the combined effect and the interaction of the compounds that are part of the tissue [11].

According to the current vision of “The Plan List”, *Hemerocallis citrina* Baroni (Xanthorrhoeaceae family), commonly known as daylily, has demonstrated multiple functions, such as antioxidant [12], sleep promoting [13], and antidepressant [14]. As recorded in the Compendium of Materia Medica, the most comprehensive and complete medicine in the history of traditional Chinese medicine, the daylily has the function of relieving gloom, and it was named ‘Wang you cao’ [15]. In recent years, some researches have demonstrated that the use of the daylily in patients between 11 to 80 years old relieved the depressive symptoms [16]. Moreover, studies performed in vivo demonstrated that the antidepressant effect of daylilies was related to the reversion of monoamine neurotransmitter dysfunction, which could increase the 5-hydroxytryptamine (5-HT), norepinephrine (NA), and dopamine (DA) levels in mice brain [15,17,18,19]. Aside from its antidepressant function, the facilitation of the daylily in lactation has also been recorded in plenty of ancient traditional Chinese medicine books, including the Compendium of Materia Medica, Common folk herbs in Kunming and Dian Nan Materia medica. Parturient women living in daylily growing areas mostly use it as food to promote milk secretion and they consume it in different ways, such as stew with fish, cooked porridge with pork, and they fry it alone [20,21]. A previous study has demonstrated that the use of daylily as functional food improved the offspring’s weight gain and serum PRL level in dams [22]; however, the molecular mechanism of the daylily on lactation remains unknown and needs to be elucidated.

With the development and improvement of omics technology, it has been acknowledged that the complex mechanism of drugs acting against diseases often consists of treating and regulating multiple targets [23]. In order to better explore the complex relationship between drugs and diseases, Hopkins [24] proposed the concept of network pharmacology, which is based on the theory of biological systems, understanding the interaction between drugs and organism from the perspective of improving or restoring the balance of the biological network. Compared with the traditional pharmacology, network pharmacology pays more attention to medicinal interaction and to the integrity of drugs’ action on the human body. This research strategy has the same goal as the principle of multi-component, multi-target, and multi-channel synergy of diet therapy. In recent years, network pharmacology has been applied to explore the active ingredients, action targets, and the related mechanisms of plant extracts [25,26,27], which not only improves the efficiency, but also enables us to learn more about the effects of plants against diseases. 

The purpose of this research was to demonstrate the usefulness of network pharmacology to explore and to study the active components, targets, and molecular mechanisms of daylilies and their effect against lactation insufficiency. Moreover, this research aimed to obtain a scientific explanation in order to validate the contents recorded in ancient traditional Chinese medicine books regarding performing experiments with animals.

## 2. Materials and Methods

### 2.1. Chemicals and Reagents

Dried flowers of *Hemerocallis citrina* Baroni were provided by Qidong Yunxing Lake Modern Agricultural Science and Technology Ecological Park Development Co., Ltd. (Qidong, Hunan, China). Anhydrous ethanol was purchased from Damao chemical reagent factory (Tianjing, China). Methanol and formic acid were purchased from Macklin (Shanghai, China). PRL and OT kit were purchased from Ruifan biotechnology company (Shanghai, China). Total RNA Kit was purchased from GBCBIO Technologies Inc. (Guangzhou, Guangdong, China). PrimeScriptTM RT reagent Kit with gDNA Eraser and TB Green® Premix Ex Taq™ II (Tli RNaseH Plus) were purchased from Takara (Beijing, China).

### 2.2. Preparation of HCE Extracts

Dried flowers of daylily were stored at −20 °C until used. For extracts’ preparation, 100 g of daylily were extracted three times with 1000 mL of 95% ethanol at 80 °C for 2 h. The infusion was cooled to room temperature, centrifuged at 10,000× *g* for 10 min at 4 °C (5417R, Eppendorf AG, Hamburg, Germany), and then concentrated in a vacuum environment by a rotary evaporator (N-1001, EYELA, Tokyo, Japan). Lastly, the concentrated solution was lyophilized for 72 h to obtain the HCE powder.

### 2.3. Chromatography and MS

UPLC-Q-Orbitrap-MS (Thermo Fisher Scientific, Bremen, Germany) combined with database of Orbitrap traditional Chinese medicine library (OTCML) were used to detect chemical constituents in HCE. The mobile phase consisted of 0.2% (vol/vol) formic acid in water (solvent A) and acetonitrile (solvent B). A HPH C18 column (2.1 × 100 mm, 4 µm) (Agilent Technologies Inc., Santa Clara, CA, USA) was employed for the separation and identification using a gradient elution program as follows: 0–12 min, 5–20%B; 12–18 min, 20–40%B; 18–20 min, 40–70%B; 20–22 min, 70–5%B. The injection volume was 5 μL. The column temperature was maintained at 40 °C, and the flow rate was 0.3 mL min−1. Mass spectrometry detection was performed in simultaneous scanning of positive and negative ion modes. Scanning range (m/z) was 100~1200. The spray voltage was 3.2 kV, and the ion source and capillary temperature were 320 °C and 350 °C, respectively. The pressure of sheath gas and assistive device were 35 arb and 10 arb, respectively [28].

Samples were prepared as follows: methanol solution was added to 300 mg of HCE powder in order to reach a final volume of 25 mL. The solution was homogenized using ultrasound for 30 min, and then, it was centrifugated at 12,000× *g* rpm for 10 min. Finally, the supernatant was filtered using a 0.2-μM filter membrane for compound detection [29].

### 2.4. Experiments Performed with Animals

Sprague Dawley rats (aged 8–9 weeks, weighing 250–280 g) were obtained from the Experimental Animal Center of Southern Medical University (Guangzhou, Guangdong, China; Certificate No. SCXK (yue) 2016–0041) and they were kept under a specific pathogen free (SPF) environment, in a dark/light cycle of 12 h/12 h in the Experimental Animal Center of South China Agricultural University (Certificate No. SYXK (yue) 2019–0136; Ethics approval number: 2020b025). All rats had ad libitum access to food and sterile water during the first week of acclimation. After acclimation, female rats were matched with male rats at 8:30 p.m. and the presence of vaginal plug, which indicates day zero of pregnancy, was inspected the next day at 8:00 am. Then, pregnant rats were divided into the following four groups: the control group (CON, *n* = 8), the chronic unpredictable mild stress group (MOD, *n* = 8), the HCE low dose group (HCEL, *n* = 8), and the HCE high dose group (HCEH, *n* = 8). Rats in the control group were fed with distilled water. Rats in CUMS group were exposed to chronic unpredictable mild stress (CUMS) and fed with 10 mL of 0.8% CMC-Na solution. Rats in HCEL group were exposed to CUMS and fed with 10 mL of HCE low dose extract solution, which was composed of HCE powder and 0.8% CMC-Na. Rats in HCEH group were exposed to CUMS and fed with 10 mL of HCE high dose extraction solution, which was composed of HCE powder and 0.8% CMC-Na. After drinking up the 10 mL solution, rats in these three groups were fed only with distilled water. The weight of rats would change during the trial, and rats in HCEL and HCEH groups were fed with 10 mL HCE extraction solution, but the concentration of HCE in 10 mL was adjusted according to the weight of female rats and based on 1.375 g/kg body weight (BW) and 5.5 g/kg BW, respectively. Animal experiments were processed with the approval of the Laboratory Animal Ethics Committee of South China Agricultural University and all procedures followed National Institutes of Health guide (NIH 85 Publications No. 8023, revised 1978).

### 2.5. Stress Procedure

From gestational day 14 to weaning, all female rats, except the control group, were exposed to CUMS (different stress separated by at least 2 h break), including wet/no bedding for 12 h, restraint stress for 30 min, squeezing tail for 5 min, exposure to foreign object for 7 h, etc. Therefore, the female rats were exposed to 8 different kind of stress situations [2,30]. Details are showed in Table S1.

### 2.6. Sample Collection

Pups in different groups were weighted at 8:00 a.m. throughout the lactation period to determine the weight difference of pups among the four different groups. On the last day of lactation, dams were anesthetized with pentobarbital sodium and blood samples were collected by performing abdominal aorta puncture. The mammary gland tissue from the left abdomen of the female rats were fixed with 4% paraformaldehyde for preparing paraffin section, and the right breast tissue was stored at −80 °C refrigerator for further analysis of gene expression.

### 2.7. Evaluation of Lactation Promoting Activity

#### 2.7.1. Weight Changes of Pups

At 8:30 every morning, the pups were taken out of the cage, and the weight of each litter was weighed and recorded for 21 days.

#### 2.7.2. Biochemical Analyses

The blood samples collected from dams were centrifuged at 3000× *g* rpm for 20 min at 4 °C to get the serum and it was stored at −80 °C. PRL and OT levels of the serum were determined by using an ELISA detection kit (Meimian Biotech, Shanghai, China).

#### 2.7.3. Histopathological Analysis

The mammary gland tissue of the dams was fixed in 4% paraformaldehyde for 24 h and then embedded in paraffin for sectioning. The section was stained with hematoxylin–eosin. Pathological changes in the tissues were observed through optical microscope (Carl Zeiss AG, Jena, Germany) under 100 and 200 times magnification.

### 2.8. Analysis of Potential Mechanism

#### 2.8.1. Screening Targets of HCE–Lactation

Online databases were used to obtain the targets of HCE, including Traditional Chinese Medicine Systems Pharmacology (TCMSP, http://tcmspw.com), PubChem (https://pubchem.ncbi.nlm.nih.gov/), and SwissTargetPrediction (http://www.swisstargetprediction.ch/). At the same time, by the use of “lactation” as a key word to search for disease targets in database, including GeneCard (https://www.genecards.org/), Drugbank (https://www.drugbank.ca/) and OMIM (https://omim.org/). After that, all primary targets were input into jvenn (http://jvenn.toulouse.inra.fr/) to obtain the interaction targets of HCE extraction and lactation. Lastly, the common targets of daylily and lactation were input into Cytoscape 3.7.2 in order to construct the “drug-targets-disease” network.

#### 2.8.2. PPI Network Construction

In order to analyze the relationship between daylily and milk secretion targets, the protein-protein interaction (PPI) network of intersection targets was constructed by the STRING (https://string-db.org/) database version 11.0. The minimum interaction threshold was set to 0.9 (high confidence), and then, the constructed PPI network was imported into Cytoscape 3.7.2 software for network topology analysis by the use of a plugin named NetworkAnalyzer.

#### 2.8.3. KEGG Enrichment Analysis

In order to explore the molecular mechanism of daylily promoting lactation, Metascape (http://metascape.org/gp/index.html) was used for KEGG enrichment analysis. Then, Cytoscape 3.7.2 was used for KEGG pathway clustering analysis.

### 2.9. Total RNA Extraction and Real Time PCR

Total RNA was isolated from the mammary gland of dams by using the Total RNA Kit. The purity and concentration of RNA were measured by Nanodrop ultramicro spectrophotometer (Thermo Fisher Scientific, Waltham, MA, USA). cDNA was synthesized from 1 μg of RNA using the PrimeScriptTM RT reagent Kit. The mRNA expression was performed using a TB Green^®^ Premix Ex Taq™ II kit and Bio-Rad C1000 Thermal Cycler Real-Time PCR System (Bio-Rad, Hercules, CA, USA). Relative mRNA expression was measured by relative quantification with β-actin as the internal reference. The primer sequences of all the targets are shown in Table S2. Relative gene expression was calculated according to the 2^−ΔΔCt^ method.

### 2.10. Statistical Analysis

Data are expressed as mean ± standard deviation (SD) and were analyzed by SPSS 21.0 (SPSS Inc., Chicago, IL, USA). The significant differences between groups were calculated using one-way factorial analysis (ANOVA) followed by the least significant difference (LSD). The *p*-value of <0.05 was considered statistically significant. The networks were constructed though Cytoscape 3.7.1 (Boston, MA, USA) combined with the ClueGo and CluePedia package. Other bar charts in this study were constructed by GraphPad Prism7 (GraphPad, Inc., San Diego, CA, USA).

## 3. Results

### 3.1. Identification of the Components in HCE Based on UPLC-Q-Orbitrap-MS and OTCML

Results showed that 70 different compounds were detected in HCE, including 27 flavonoids, 7 phenolic acids, 7 sugars, 6 amino acids, 4 coumarins, 2 alkaloids, and 17 other types of compounds. Details are described in Table S3 and Figure S1.

### 3.2. Effects of HCE on Lactation of CUMS Dams

#### 3.2.1. Pups Weight Change

**Figure 1:**
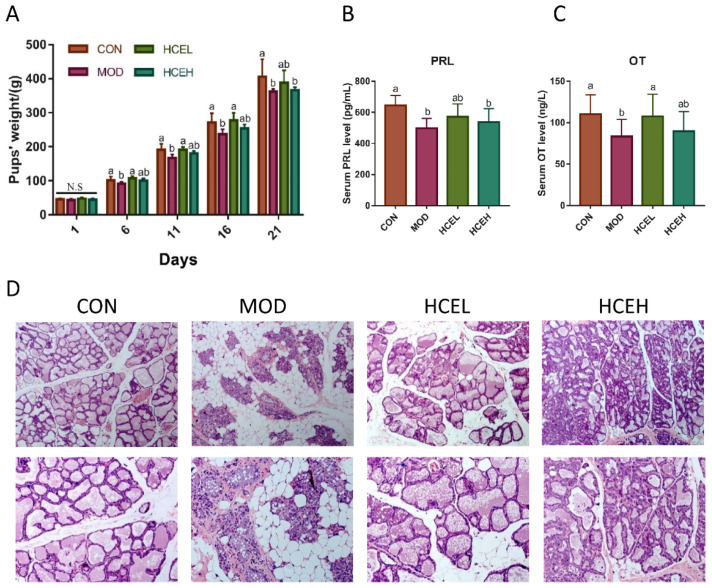
Effects of HCE on lactation of CUMS dams. (**A**) Pups’ weight change during the lactation. (**B**) Serum PRL level of female rats among four groups. (**C**) Serum OT level of female rats among four groups. (**D**) Histopathological analysis of the mammary gland sections of rats in different groups at 10× and 20× magnification. For images A–C, different letters indicate a significant difference between columns, *p* < 0.05. CON: the control group (*n* = 8); MOD: the chronic unpredictable mild stress group (*n* = 8); HCEL: the HCE low dose group (*n* = 8); HCEH: the HCE high dose group (*n* = 8).

Throughout the lactation period, the body weight of pups changed. As illustrated in Figure 1A, as a general trend, the pups’ weight rose in all groups, with a weight gain different between them. On the first day of birth, there was no significant difference in pups’ body weight in either of the four groups. However, on day 6th, a remarkable difference in pups´ body weight was observed in relation to the pups of dams in CUMS which showed a lower body weight than that of the control group. Moreover, the body weight of offsprings in the two groups supplemented with HCE was higher than that of dams in CUMS groups, while the HCEL group showed a significantly higher difference. These changes remained until the weaning. On the last day of lactation, in comparison with the CUMS group, the pups’ average weight gain in the HCEL group was increased by 7.18%.

#### 3.2.2. Serum PRL and OT Level

As shown in Figure 1B,C, the serum PRL and OT levels of maternal rats in the control group was significantly higher than those in the CUMS group (*p* < 0.01), indicating that CUMS might inhibit the secretion of PRL and OT. Interestingly, after supplementation with HCE, the levels of serum PRL and OT in CUMS dams were increased, and the OT level showed a remarkable difference.

#### 3.2.3. Mammary Gland Tissue of Dams

As Figure 1D shows, in the control group, the ducts extended throughout the breast tissue with irregular shaped acini which contained secretion around it consist of a network of mammary gland flocculus. However, there was almost no acini of dams in the CUMS group, where acinus cavity was smaller than the control group, and was surrounded by a large number of adipocytes and connective tissue, showing the degeneration of the mammary gland function. After supplement with HCE, the mammary gland of dams in the HCEL and HCEH groups took a favorable turn with less connective tissue, thicker ducts, as well as more secretion. 

### 3.3. Analysis and Validation of Potential Therapeutic Mechanism of HCE

#### 3.3.1. Potential Target Screening of HCE by Network Pharmacology

**Figure 2:**
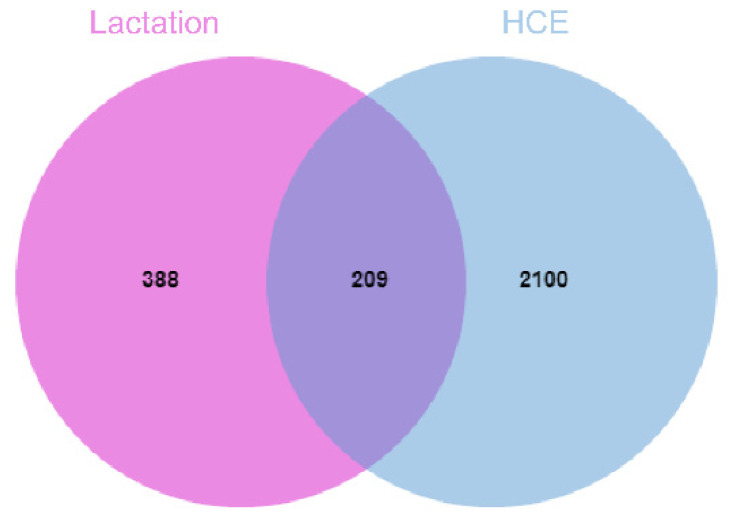
Venn diagram of lactation and daylily.

Through an online database, a total of 2309 targets (Table S4) were obtained by using the name of the 70 detected compounds in the daylilies and 597 disease targets (Table S5) using ‘lactation’ as keyword. Finally, 209 common targets were obtained through jvenn (Figure 2), which indicates that these 209 targets might be associated with the used of daylilies in lactation insufficiency treatment.

#### 3.3.2. Construction of “HCE-Targets-Lactation” Network

**Figure 3:**
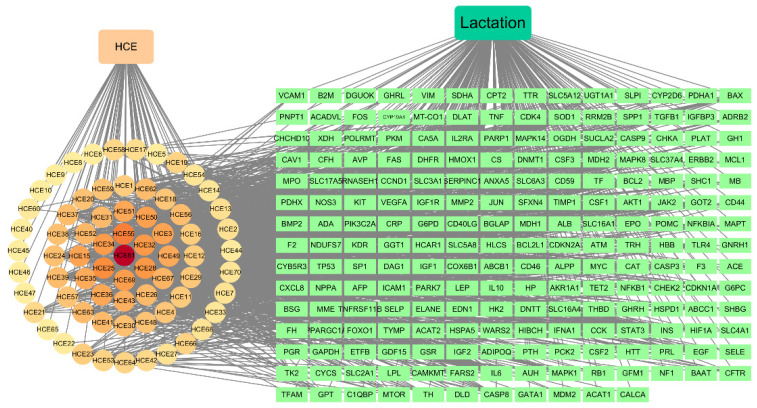
HCE-targets-lactation network. Circular nodes: compounds of daylily; Square nodes: common targets of daylily and lactation.

After inputting the compounds, common target, and lactation into the Cytoscape software, the “HCE–targets–lactation” network was constructed (Figure 3). The topological analysis results (Table 1 and Table S6) of this network indicated that from the 68 detected compounds, 62 were linked with targets of lactation. Among them, quercetin (HCE61), kaempferol (HCE55), thymidine (HCE25), chlorogenic acid (HCE28), caffeic acid (HCE32), and rutin (HCE34) were the six compounds most connected with the largest number of lactation targets, indicating that these constituents might be the most important material basis of daylilies for promoting lactation.

#### 3.3.3. PPI Network of HCE-Lactation

**Figure 4:**
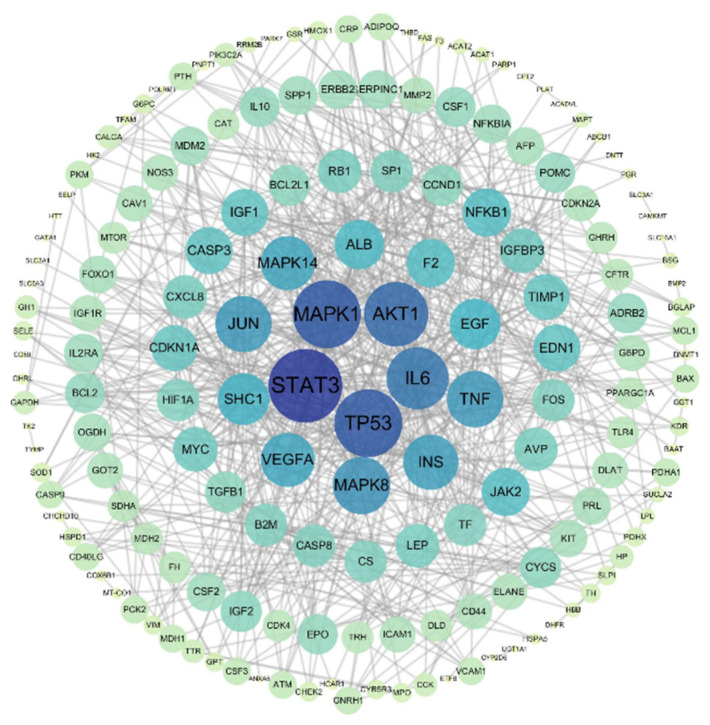
PPI network of common targets between daylily and lactation.

In order to understand the relationship between the 206 common targets, a PPI network was constructed through STRING and Cytoscape 3.7.2. As Figure 4 shows, 206 number of nodes and 692 number of edges were contained in the PPI network, in which the bigger the node, the darker the color, which means a strong interaction between targets. The top 8 targets of this network were STAT3 (degree = 41), MAPK1 (degree = 37), TP53 (degree = 35), AKT1 (degree = 33), IL6 (degree = 41), MAPK8 (degree = 31), TNF (degree = 27), and JUN (degree = 26), indicating that these targets were the core genes of daylilies for promoting lactation.

#### 3.3.4. KEGG Enrichment Analysis

**Figure 5:**
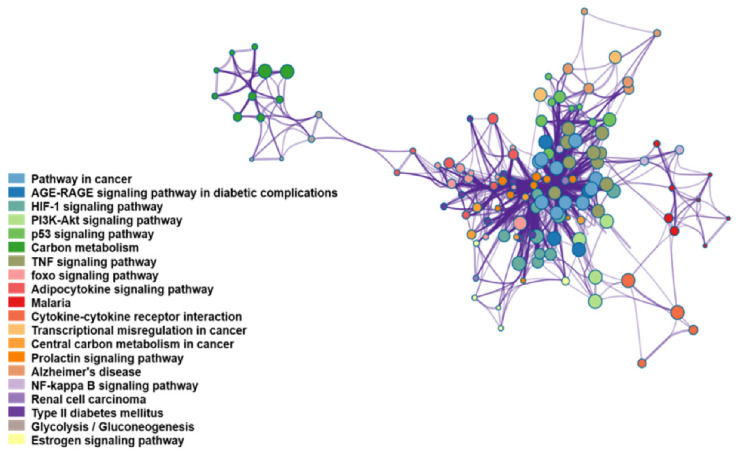
The result of KEGG pathway enrichment analysis.

In order to elucidate the molecular mechanism of daylilies promoting lactation, 206 targets were selected for KEGG enrichment analysis and then, all pathways were analyzed by a cluster analysis. Results are shown in Figure 5, where 20 types of pathways related to daylily promoting lactation have been found, which are mainly involved with the immune system, endocrine system, and signal transduction. Among these pathways, prolactin signaling pathway is the most important one, containing 19 of 20 core targets and the 18.57% of the genes on this pathway were associated with HCE. Based on these results, this pathway was selected to be verified in animal experiments.

#### 3.3.5. Gene Expression of Prolactin Signaling Pathway

**Figure 6:**
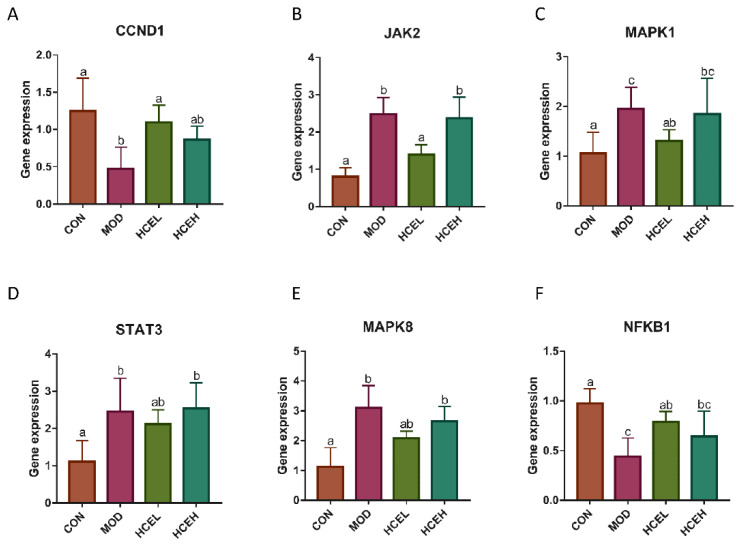
**The** effects of HCE on gene expression of prolactin signalling pathway in mammary gland of dams. The expression of CCND1 (**A**), JAK2 (**B**), MAPK1 (**C**), STAT3 (**D**), MAPK8 (**E**), and NFKB1 (**F**). For all images, different letters indicate a significant difference between columns, *p* < 0.05. CON: the control group (*n* = 5); MOD: the chronic unpredictable mild stress group (*n* = 5); HCEL: the HCE low dose group (*n* =5); HCEH: the HCE high dose group (*n* = 5).

As Figure 6A–F shows, the expression of NFKB1 and CCND1 in the mammary gland tissue of dams in the HCEL and HCEH groups was higher than those in the CUMS group, with HCEL being the group that showed the significantly higher difference. On the other hand, the expression of MAPK1, MAPK8, STAT3, and JAK2 in the mammary gland tissue of dams in the HCEL and HCEH groups was lower than those in the CUMS group, and the HCEL group showed a significantly lower difference.

## 4. Discussion

This research represents the first study where the benefits and the impact of daylily in promoting milk secretion has been demonstrated, by using scientific methods exploring and explaining the mechanisms through network pharmacology in a comprehensive and systematic way. Firstly, a network of “drug-targets-disease” was constructed and 62 compounds from daylily were identified as milk promoting. In addition, 260 pathways and 206 targets were identified through PPI network and KEGG enrichment. Furthermore, the specific changes of the daylily on lactation defect were evaluated through animal validation tests. Finally, by using the network pharmacology and the animal validation experiment, the benefits of the daylily on lactation and its multi-component, multi-target molecular mechanism was revealed.

Based on the ‘drugs–targets–disease’ network, the results of screening the bioactive compounds of daylily showed that a total number of 62 compounds positively affect breast milk secretion, with quercetin, kaempferol, thymidine, chlorogenic acid, caffeic acid, and rutin being the most important compounds. The published literature has indicated that quercetin initiated the lactation process by promoting the prolactin receptor (PRLR) expression and increased the expression of β-casein, stearoyl-coenzyme a desaturase, fatty acid synthase, and α-lactalbumin in mammary tissue, which are responsible for the production of fatty acids, lactose, and galactose in milk [31]. Rutin promotes the regulation of hormone secretion associated with the development of breast tissue, such as estrogen, growth hormone, and PRL [32]. This effect may be related to the ability of its metabolites to bind to estrogen receptors and produce estrogen-like effects [33]. Moreover, neurotransmitters are an important factor in lactation homeostasis, acting on mammary epithelial cells through an autocrine–paracrine mechanism to influence lactation and milk composition [34]. There are several compounds in HCE that have a modulating effect on neurotransmitters, including those in [35,36,37], which might indirectly regulate lactation. Thus, we speculated that the facilitation of the daylily on lactation is not only due to the effect of a single compound, but also the result of the interaction between several compounds. Additionally, through network pharmacological analysis, we have identified a number of compounds with potential lactation-promoting activity. As shown in Figure 3, kaempferol, thymidine, and chlorogenic acid were linked to multiple genes in the mTOR signaling pathway, including AKT1, MAPK14, and TP53, etc. A previous study has used animal models and suggested that the mTOR signaling pathway had a role in regulating milk protein synthesis [38]. The review then detailed the role of this signaling pathway in the regulation of energy, amino acid, and amino acid transport during lactation synthesis [39]. The determination of whether these compounds are important for milk protein synthesis requires further testing.

Milk secretion is an extremely complex physiological process, where fully developing mammary gland, normal hormone level, as well as other local factors are essential for successful milk secretion [40]. Network pharmacology prediction results showed that, among the common targets between daylily and lactation, there were several targets involved in regulation of breast involution [41], including STAT3, MAPK1, and IL 6, and some targets with the function of the functional differentiation of mammary gland [42,43], such as CCND1, PRLR, and TGFB1, as well as targets which have ability of regulating the biosynthesis of milk components [39], including AKT1, TF, and INS, etc. Furthermore, quercetin has been reported to promote milk secretion by significantly increasing PRLR levels [31]. Kaempferol has a significant potential to inhibit STAT3 and IL6 expression [44]. This suggests that the mechanism of daylily for promoting lactation is realized through the regulation of multiple targets and by a variety of compounds.

Enrichment analysis indicated that the mechanism of daylily extract function lactation was associated with multiple biological processes, including immune system, endocrine system, and signal transduction. Prolactin signaling pathway is the most important pathway in lactation, which can be activated by prolactin, and its most representative role is the ability to induce lobular epithelial growth and to stimulate postpartum milk secretion [45]. Animal validation tests showed that there was a significant difference in serum PRL level between the CON group and CUMS group. Considering this result, we further studied the gene expression of this pathway. The results showed that HCE supplementation altered the expression of STAT3, MAPK1, MAPK8, NFKB1, CCND1, and JAK2, indicating that HCE improved the abnormal expression of genes in the prolactin signaling pathway. CCND1 is the main downstream protein of prolactin signaling; deletion of this gene brings about a dearth of alveolar cell and failure of functional differentiation as well as lactation defect [22,46,47]. MAPKS induced the inflammation of mammary gland and the apoptosis of mammary epithelial cells [48], and then, affecting the milk components synthesis [49]. Compared with HCE-fed dams, the expression of CCND1 decreased and the expression of MAPK1 and MAPK8 were increased in CUMS dams, which induced the premature involution of breast in dams of lactation, manifesting as more fat and connective tissue, and lower body weight gain in their pups.

In previous studies on promoting lactation, the gene expression of JAK2 was significantly decreased in a lactation deficient group, while our experiment showed an opposite results. It could be assumed that the main reasons for this behavior is that the mammary gland has entered in the remodeling stages, JAK2 no longer activates STAT5 to promote milk fat synthesis like lactation stage, but activates STAT3, accelerating the functional degeneration of the mammary gland [50]. In addition, in mammary involution of healthy mice, the gene expression of NF-κB undergoes the process of first increasing and then decreasing, usually rising to the highest level on three days after weaning [51,52]. Thus, the expression of NFKB1 in the CUMS group was significantly lower than that in the other groups, which might be due to the fact that the mammary glands of the CUMS group tended to the original state gradually, while other groups were still in the initial stage of degeneration.

## 5. Conclusions

In the present study, we confirmed that the daylily can improve milk secretion though regulating multiple targets and pathways. The prediction results of network pharmacology indicated that there were 62 compounds in the HCE that could regulate lactation targets, among them, quercetin, kaempferol, and another five compounds were the important material basis. The mechanism of promoting lactation was related to the regulation of 205 targets and 260 pathways. Subsequent animal validation tests showed that, after supplement with HCE, the symptoms of lactation insufficiency in CUMS female rats were significantly improved, manifesting as the higher weight gain of pups, and the significant increase of serum PRL and OT levels. Besides, the daylily slowed down the degradation of the lactation function in the mammary gland of CUMS dams by regulating the expression of multiple genes in the prolactin signaling pathway. This study not only scientifically explains the traditional saying that the daylily promotes lactation, but also provides an efficient method for the study of plant efficacy.

## Figures and Tables

**Table 1:** The topology analysis of top 6 compounds in “HCE-targets-lactation”.

No.	Name	Degree	Betweenness Centrality	Closeness Centrality
HCE61	Quercetin	95	0.09	0.49
HCE55	Kaempferol	52	0.02	0.42
HCE25	Thymidine	47	0.02	0.42
HCE28	Chlorogenic acid	42	0.01	0.41
HCE32	Caffeic acid	41	0.01	0.41
HCE34	Rutin	40	0.01	0.41

## Data Availability

Data are contained within the article or supplementary material.

## Supplementary Materials

The following are available online at https://www.mdpi.com/article/10.3390/foods10081863/s1, Figure S1: Chromatograms of compounds in HCE, Table S1: Example of the chronic unpredictable mild stress paradigm during perinatal period, Table S2: Sequences of primer used in real time PCR, Table S3: Representative MS fragments of the compounds of 95% ethanol extracts from HCE, Table S4: Targets of HCE cpmpounds, Table S5: Targets of lactation, Table S6: Topology analysis of compounds in “HCE-targets-lactation”.
