# Supplementary material for: Study and Experimental Validation of the Functional Components and Mechanisms of Hemerocallis citrina Baroni in the Treatment of Lactation Deficiency
Source: Foods. 2021 Aug 12;10(8):1863. doi: 10.3390/foods10081863 (PMC8391212; doi:10.3390/foods10081863)
Supplement: Supplementary file 1 [file foods-10-01863-s001.zip › Figure S1 Chromatograms of compounds in HCE.pdf]

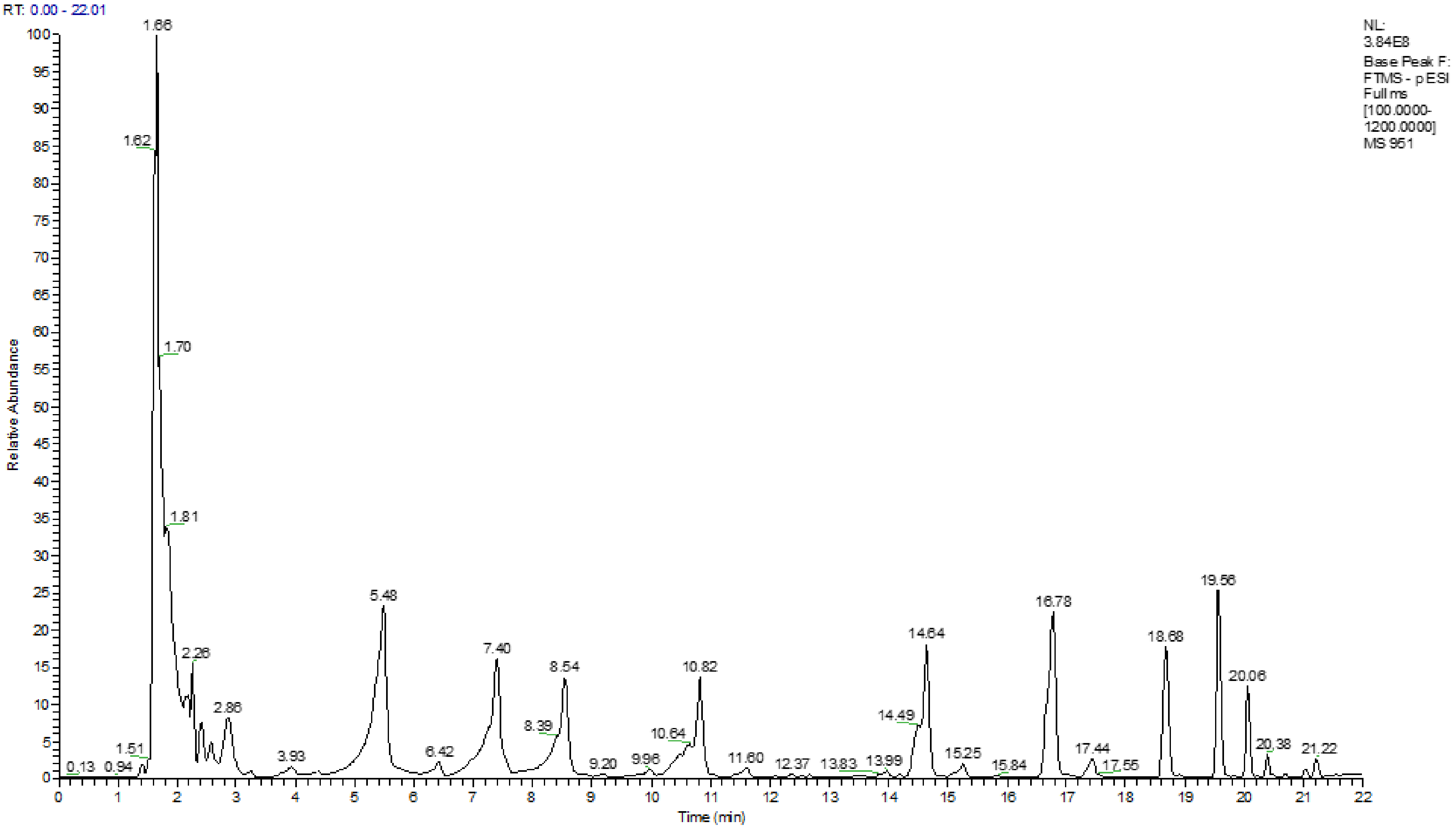


**Figure S1** Chromatograms of compounds in HCE by UPLC-Orbitrap-MS in ESI-.

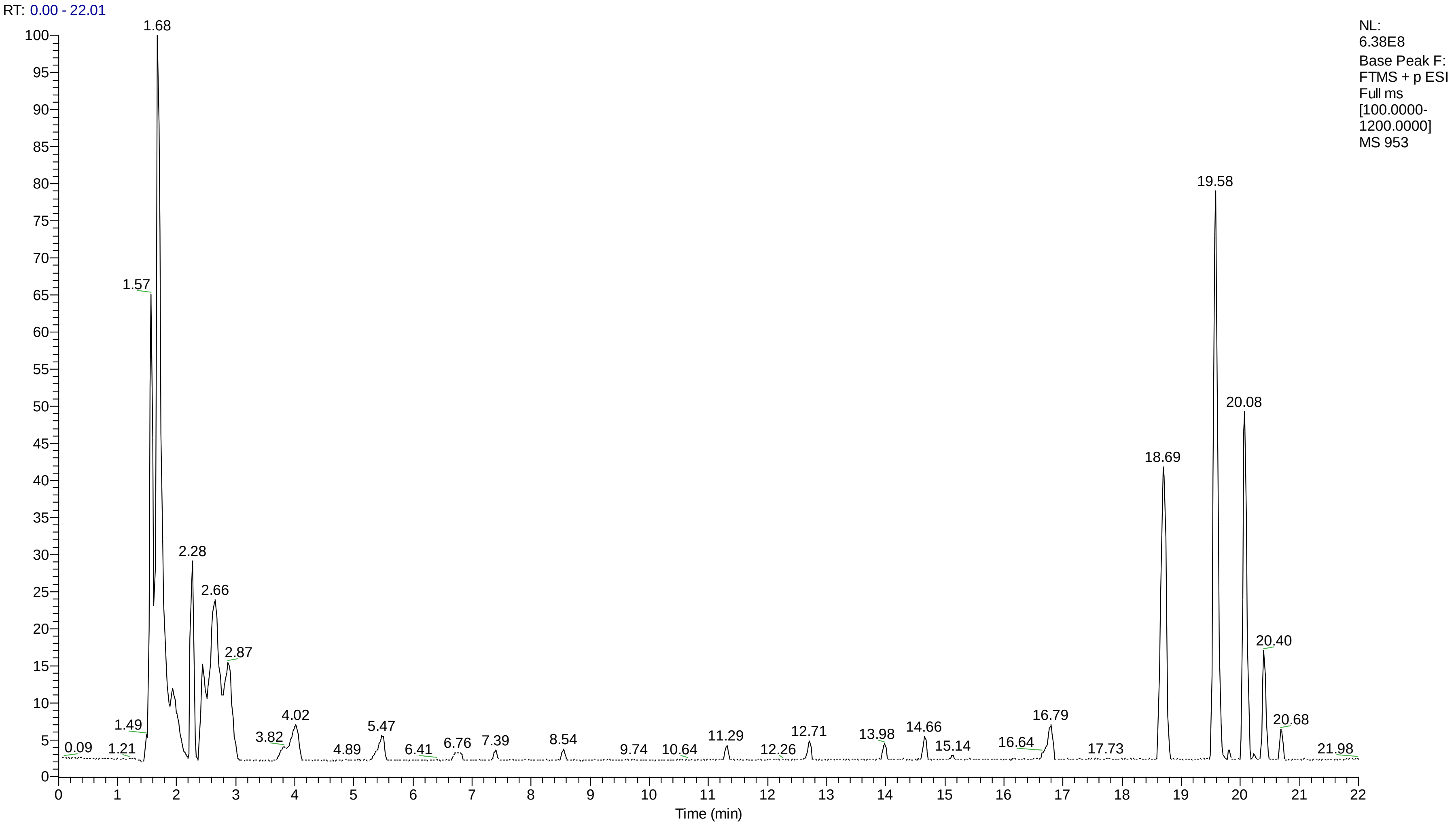


**Figure S2** Chromatograms of compounds in HCE by UPLC-Orbitrap-MS in ESI+.
