# Supplementary material for: Study and Experimental Validation of the Functional Components and Mechanisms of Hemerocallis citrina Baroni in the Treatment of Lactation Deficiency
Source: Foods. 2021 Aug 12;10(8):1863. doi: 10.3390/foods10081863 (PMC8391212; doi:10.3390/foods10081863)
Supplement: Supplementary file 1 [file foods-10-01863-s001.zip › Table S1-2 Example of the chronic unpredictable mild stress paradigm during perinatal period..pdf]

Table S1 Example of the chronic unpredictable mild stress paradigm during perinatal period.

Day	Stress (beginning time)	Day	Stress (beginning time)
GP 14th	Wet bedding 12 h (8:30)	GP 15th	Restraint stress 30 min(10:00)
GP 16th	No bedding 12 h (20:00)	GP 17th	Cage tilt 45°,12 h (20:00)
GP 18th	Restraint stress 30 min (10:00)	GP 19th	Squeezing tail 5 min (8:30)
GP 20th	No stress	GP 21th	No stress
Delivery	No stress	PP 1st	No stress
PP 2nd	Restraint stress 2 h (8:30)	PP 3rd	25 °C swimming 20 min (8:30)
PP 4th	Wet bedding 12 h (8:30)	PP 5th	Squeezing tail 5 min (8:30)
PP 6th	Depriving food 12 h (20:00)	PP 7th	Exposure to foreign object 7 h (9:00)
PP 8th	Restraint stress 2 h (8:30)	PP 9th	Cage tilt 45° 12 h (20:00)
PP 10th	Squeezing tail 5 min(8:30)	PP 11th	25 °C swimming 20 min (8:30)；<br>wet bedding 12 h (20:00)
PP 12th	Exposure to foreign object 7 h (9:00)	PP 13th	4 °C swimming 5 min(8:30)
PP 14th	25 °C swimming 20 min (8:00)	PP 15th	Restraint stress 2 h (8:30),<br>squeezing tail 5 min(10:30)
PP 16th	No bedding 12 h (20:00)	PP 17th	Wet bedding 12h (8:30)
PP 18th	4 °C swimming 5 min (8:30)	PP 19th	Depriving food 12 h (20:00)
PP 20th	Restraint stress 2 h (8:30)	PP 21st	No bedding 12 h (20:00)

Notes: GP, gestation period; PP, postpartum period.

Table S2 Sequences of primer used in real time PCR

Genes	Forward Primer	Reverse Primer
STAT3	5′-GCAGAAGACACTGACCGATGAAGAG-3′	5′-CCACGATCCTCTCCTCCAGCATT-3′
CCND1	5′-GAGGCGGATGAGAACAAGCAGATC-3′	5′-GTGCGGTAGCAGGAGAGGAAGT-3′
NFKB1	5′-CCAGCACCAAGACCGAAGCAAT-3′	5′-GCCAGCAGCATCTTCACATCTCC-3′
MAPK1	5′-CCTTCCAACCTCCTGCTGAACAC-3′	5′-GCGTGGCTACATACTCTGTCAAGAA-3′
MAPK8	5′-GGAGCGAACTAAGAATGGCGTCAT-3′	5′-TCATCTACAGCAGCCCAGAGGTC-3′
JAK2	5′-AGAATGTCTTGGCATGGCAGTGTT-3′	5′-ATCTTCGCTCGCACGCACTTC-3′
β-actin	5′-CACTGCCGCATCCTCTTCCT-3′	5′-AACCGCTCATTGCCGATAGTG-3′
